# Prenatal allostatic load and adverse pregnancy outcomes: a systematic review and meta-analysis

**DOI:** 10.7717/peerj.21756

**Published:** 2026-09-24

**Authors:** Mengjie Zhao, Hanyue Zheng, Dengyan Qi, Xuening Zhang, Lei Ba, Bei Wang, Xiang Hong

**Affiliations:** 1 Key Laboratory of Environmental Medicine Engineering, Ministry of Education, School of Public Health, Southeast University, Nanjing, China; 2 National Health Commission Key Laboratory of Contraceptives Vigilance and Fertility Surveillance, Nanjing, China; 3 Jiangsu Provincial Medical Key Laboratory of Fertility Protection and Health Technology Assessment, Nanjing, China; 4 Jiangsu Health Development Research Center, Nanjing, China

**Keywords:** Allostatic load, Maternal, Preterm birth, Preeclampsia, Meta-analysis

## Abstract

**Background:**

Allostatic load (AL) denotes the cumulative physiological dysregulation resulting from chronic stress exposure. This systematic review and meta-analysis aimed to evaluate the association between prenatal AL and adverse pregnancy outcomes (APOs), and to synthesize current evidence to inform prenatal health strategies and potentially reduce the incidence of APOs.

**Methodology:**

This study systematically searched PubMed, Web of Science, Embase, PsycINFO, China National Knowledge Infrastructure, Wanfang, VIP China Science and Technology Journal Database, and SinoMed databases for relevant studies examining prenatal AL and APOs published from September 1993 to 22 June 2026. Two researchers independently screened the literature, extracted data, and assessed the risk of bias using the Newcastle-Ottawa scale. Meta-analysis was performed using R software (version 4.4.3). The study protocol is registered in PROSPERO (CRD420251076952).

**Results:**

Eight original studies involving 8,795 pregnant women were included. High prenatal AL was associated with an increased risk of preterm birth (OR = 1.29, 95% CI [1.04–1.60]) and preeclampsia (OR = 2.31, 95% CI [1.42–3.76]). There was no statistically significant association with low birth weight (OR = 1.10, 95% CI [0.95–1.26]). Furthermore, exploratory subgroup analyses suggested that the gestational period and biomarker composition may influence these associations; however, high methodological heterogeneity limits direct clinical application.

**Conclusions:**

Elevated prenatal AL is associated with an increased risk of specific APOs, particularly preterm birth and preeclampsia. Future studies should adopt longitudinal designs with repeated AL measurements across pregnancy to clarify temporal dynamics and elucidate underlying biological mechanisms.

## Introduction

Adverse pregnancy outcomes (APOs), such as preterm birth (PTB), low birth weight (LBW), preeclampsia (PE), and small for gestational age (SGA), pose a substantial global public health challenge, with approximately 25% of pregnancies experiencing at least one major APOs (Lueth et al., 2022). PTB is the leading cause of neonatal mortality worldwide, with an incidence rate of approximately 10%, while SGA affects up to 16% of births (Campisi, Carbone & Zlotkin, 2019; Bradley et al., 2025). These outcomes substantially increase the risks of perinatal morbidity and mortality and may confer long-term developmental consequences for offspring (Gyllenhammer et al., 2025). The etiology of APOs is multifactorial, arising from complex interactions among maternal physiological status, genetic predisposition, lifestyle, environmental exposures, immune function, and infection history (Lu et al., 2021; Teede et al., 2022). Although established risk factors, such as advanced maternal age, pre-pregnancy obesity, chronic hypertension, and reproductive tract infections have been identified (Goldenberg et al., 2008), a proportion of APOs remain unexplained by conventional risk profiles.

In recent years, chronic psychosocial stress has emerged as a potentially modifiable risk factor for APOs (Vrekoussis et al., 2010). Substantial epidemiological evidence links elevated prenatal maternal stress with increased PTB risk across diverse cohorts (Cole-Lewis et al., 2014; Kornfield et al., 2022; Bergeron et al., 2023; Yamamoto et al., 2023). Similarly, heightened perceived stress is associated with PE, a severe complication threatening maternal-fetal well-being (László et al., 2013; Yu et al., 2013; Sarmasti et al., 2019; Lissåker et al., 2022; Aldinger, Abele & Kranz, 2024). Despite these associations, the biological mechanisms underlying stress-induced APOs remain incompletely characterized. The allostatic load (AL) model provides a conceptual framework for understanding how chronic stress contributes to APOs, particularly in populations lacking traditional risk factors (Morrison et al., 2013). The AL concept describes the relationship between stress and disease development based on allostasis—the organism’s ability to achieve stability through change (McEwen & Stellar, 1993; McEwen & Seeman, 1999). The stress response system reacts to environmental pressures by activating the sympathetic nervous system and the hypothalamic-pituitary-adrenal (HPA) axis. While this is an adaptive mechanism for acute stress, chronic activation results in “wear-and-tear” on the body, leading to cumulative multi-system dysregulation quantified as AL (Finlay et al., 2022; Parker et al., 2022).

Currently, several methods to calculate the allostatic load index have been used. The most prevalent is the “group allostatic load index” method, which can be calculated as a cumulative score of biomarkers across the cardiovascular, neuroendocrine, metabolic, and immune systems (Finlay et al., 2022). Given that conception and gestational maintenance require precise integration of these physiological systems, elevated AL (as measured by these physical indices) may impair reproductive function and disrupt pregnancy progression (Barrett et al., 2018). Consequently, the present study focuses exclusively on research utilizing these objective, biomarker-based measures.

While many studies have explored the association between prenatal AL and APOs, reported findings demonstrate significant heterogeneity. For instance, Wallace et al. (2013b) observed no association between AL and PTB risk, whereas Premji et al. (2025) identified a positive association. This systematic review and meta-analysis aims to synthesize current evidence on prenatal AL and APOs risk, elucidate the potential pathways through which AL influences pregnancy outcomes *via* multi-system dysregulation, and identify critical research gaps to inform future mechanistic studies and targeted preventive strategies.

## Methods

This systematic review and meta-analysis adhered strictly to Preferred Reporting Items for Systematic reviews and Meta-Analyses (PRISMA)/Meta-analysis Of Observational Studies in Epidemiology (MOOSE) guidelines (Stroup et al., 2000; Moher et al., 2010). The completed PRISMA and MOOSE checklists are provided in Supplementary 1 and Supplementary 2. The protocol was prospectively registered in the International Prospective Register of Systematic Reviews (PROSPERO) with the registration number CRD420251076952.

### Inclusion and exclusion criteria

Inclusion criteria: (1) Population: Prenatal women of reproductive age (typically 18–45 years); (2) Exposure: Objectively measured prenatal AL at any gestational stage, operationalized as a composite biomarker index spanning ≥ 2 physiological systems (neuroendocrine, immune, metabolic, cardiovascular, and anthropometry); (3) Comparison: Pregnant women with lower prenatal AL scores (*e.g.*, lowest *vs.* highest AL quantiles); (4) Outcomes: Primary APOs, including PTB (gestational week at birth < 37 weeks), PE (defined according to the American College of Obstetricians and Gynecologists criteria) (American College of Obstetricians and Gynecologists, 2013), LBW (a birth weight of less than 2,500 g), SGA (birth weight < 10th percentile using the Alexander birth weight norms); (5) Study design: Observational studies (prospective/retrospective cohort, case-control, or cross-sectional designs) with original quantitative data.

Exclusion criteria: (1) Non-English or non-Chinese publications; (2) Reviews, commentaries, and conference abstracts; (3) Studies with inaccessible full texts, insufficient outcome data, or unclear AL quantification methods.

### Literature search strategy

A comprehensive search was executed across eight electronic databases, including PubMed, PsycINFO, Embase, Web of Science, China National Knowledge Infrastructure (CNKI), Wanfang, VIP China Science and Technology Journal Database (VIP) and SinoMed. The search timeframe encompassed publications from September 1993 (when the AL model was first proposed (McEwen & Stellar, 1993) to 22 June 2026. The population, intervention, comparison, and outcomes (PICO) framework guided search term development. Search terms included allostatic load, AL, allostasis, preterm birth, low birth weight, birth outcomes, preeclampsia, and pregnancy. Boolean operators (AND/OR) and Medical Subject Headings (MeSH) were applied iteratively. The complete PubMed search strategy is provided in Supplementary 3.

### Literature screening and data extraction

The search strategy was independently conducted by Z.M. and Z.H. Study selection and data extraction were also carried out independently by the same two researchers. After removing duplicate records, titles and abstracts were screened against the predefined eligibility criteria. The full texts of potentially eligible studies were then assessed for inclusion. If there was duplicate publication or overlapping of the study populations, the study with a broader coverage of the population and more comprehensive information was selected. Any disagreements arising during the study selection or data extraction processes were resolved through discussion and consensus, or, when consensus could not be reached, through arbitration by a third reviewer (Q.D.). Data extraction included the following: Study characteristics (authors, publication year, country, design, sample size); Exposure metrics (AL calculation method, biomarkers per physiological system, trimester of assessment); Outcome definitions and effect estimates (adjusted odds ratios (ORs) with 95% confidence intervals (CIs)). To ensure the statistical independence of the data, a prespecified hierarchy was applied for data extraction. First, we confirmed that all included studies assessed outcomes at a single gestational time point. Second, when multiple analytical models were presented within a single study, we extracted the estimate from the most fully adjusted model. Finally, if the study categorized AL into more than two groups, the estimate comparing the highest AL level against the lowest was prioritized. To enhance the comparability of the AL index across studies, biomarkers were reclassified according to Juster, Mcewen & Lupien (2010) research into the neuroendocrine system (*e.g.*, cortisol, dehydroepiandrosterone sulphate), the immune system (*e.g.*, C-reactive protein, interleukin-6), the metabolic system (*e.g.*, triglycerides, high-density lipoprotein cholesterol, low-density lipoprotein cholesterol), the cardiovascular system (*e.g.*, diastolic blood pressure, systolic blood pressure, pulse), and anthropometric measurements (*e.g.*, waist-to-hip ratio, body mass index (BMI)).

### Quality assessment

Two researchers independently assessed the risk of bias in each included study using the Newcastle-Ottawa Scale (NOS) and cross-checked the results. The NOS assesses three domains: Selection (4 items: representativeness, exposure ascertainment, *etc.*); Comparability (2 items: adjustment for key confounders); Outcome (3 items: assessment method, follow-up adequacy). Scores range from 0 to 9 (stars), with ≥ 7 indicating high quality.

### Statistical analysis

Meta-analysis was conducted using R Core Team (2025). The OR was used as the combined effect measure, with 95% CI and *P*-values calculated. Heterogeneity among studies was assessed using Cochran’s Q test (significance level *P* < 0.10) in conjunction with the *I*^2^ statistic. Given the inherent clinical and methodological heterogeneity in AL measurements across studies, a random-effects model was employed a priori as the primary analysis, with fixed-effects models restricted to sensitivity analyses. Publication bias was assessed using funnel plots as an exploratory visual assessment. To explore sources of heterogeneity, we performed prespecified subgroup analyses stratified by country, trimester of pregnancy, method of AL assessment, and the system by which AL was categorized. The robustness of our pooled estimates was evaluated *via* leave-one-out sensitivity analyses. Statistical significance was defined as two-tailed *P* < 0.05.

## Results

### Study screening process

**Figure 1:**
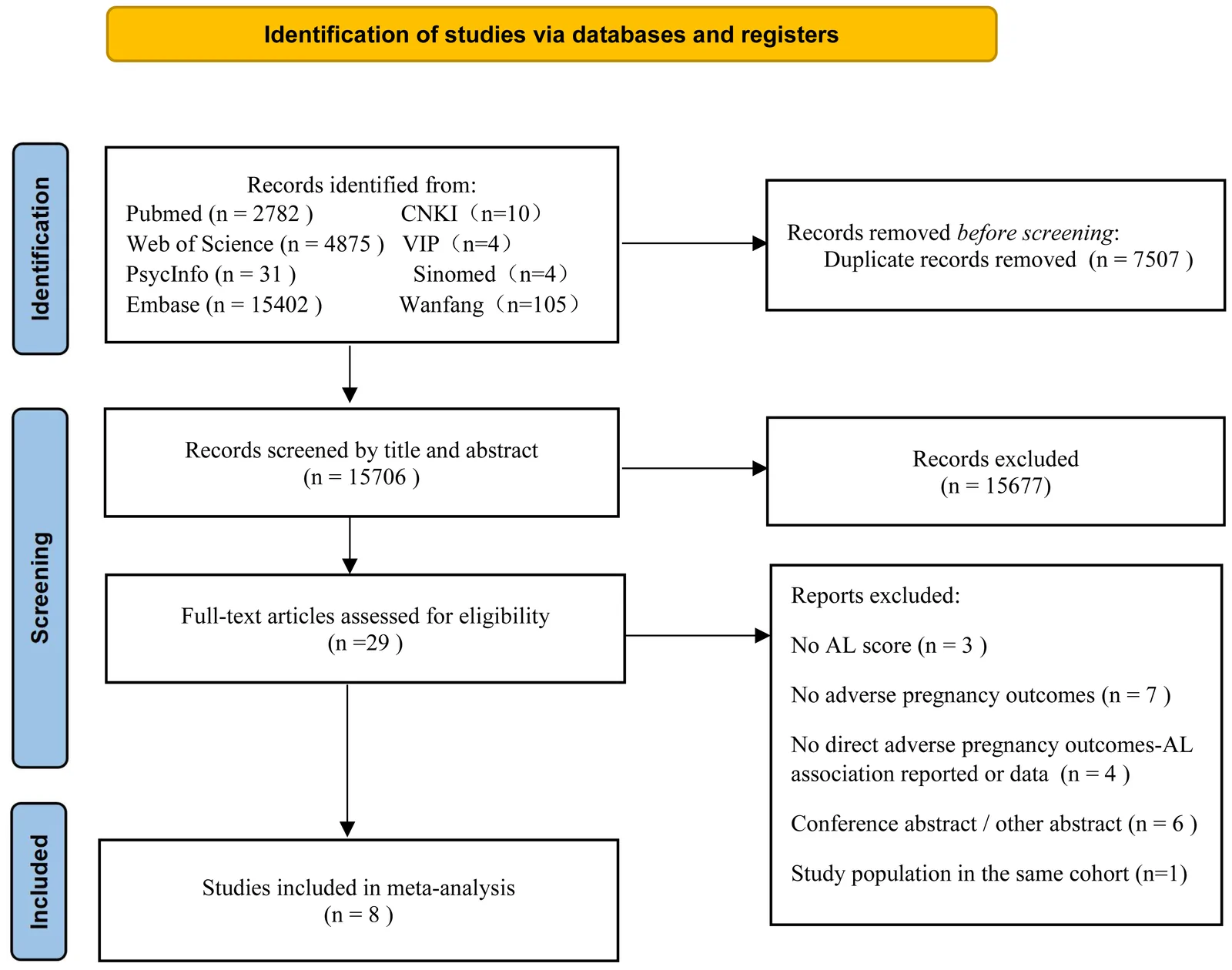
Literature screening flowchart.

Initial database searches identified 23,213 potentially relevant publications. Following the removal of 7,507 duplicate records through EndNote X20, 15,706 studies underwent title/abstract screening. Of these, 15,677 were excluded for not meeting eligibility criteria (*e.g.*, unclear AL quantification methods, irrelevant exposures). The remaining 29 publications underwent full-text assessment. During the screening process, we identified that two publications by Wallace et al. (2013a) and Wallace et al. (2013b) utilized the same underlying cohort. Because the study population of Wallace et al. (2013a) (*N* = 379) was a subset entirely contained within Wallace et al. (2013b) (*N* = 866), we retained Wallace et al. (2013b) for the final meta-analysis and excluded Wallace et al. (2013a). In total, eight studies satisfied all predefined criteria (Wallace et al., 2013b; Hux & Roberts, 2015; Barrett et al., 2018; Lueth et al., 2022; Wang, 2023; Premji et al., 2025; Zhou et al., 2025; Rittenhouse et al., 2025). The PRISMA flow diagram (Fig. 1) details the screening process with exclusion rationales at each stage.

### Basic characteristics of included studies

The included studies were published between 2013 and 2025 and encompassed 8,795 pregnant women from four countries (Table 1). Reported APOs included PTB, LBW, macrosomia, SGA, and PE. AL indices incorporated a mean of nine biomarkers representing four physiological systems. Metabolic (triglycerides, glucose), cardiovascular (blood pressure, pulse), and anthropometric (BMI, waist circumference) biomarkers were assessed in all studies, whereas neuroendocrine (cortisol, dehydroepiandrosterone sulfate) and immune markers (C-reactive protein (CRP), interleukin-6) were reported in 50% and 87.5% of studies, respectively. Most studies employed population-based risk thresholds to dichotomize biomarkers, with AL calculated as cumulative risk scores. Furthermore, while the included studies achieved relatively high numerical scores on the NOS (mean: 8; range: 7–9), substantial exposure domain-level heterogeneity remains. Specifically, the individual biomarkers comprising the AL index, as well as the methodological approaches applied, varied substantially across studies, resulting in heterogeneity in exposure definitions.

### Association between AL and APOs

Quantitative synthesis included seven studies for PTB (Wallace et al., 2013b; Barrett et al., 2018; Lueth et al., 2022; Wang, 2023; Premji et al., 2025; Zhou et al., 2025; Rittenhouse et al., 2025), four for PE (Hux & Roberts, 2015; Barrett et al., 2018; Lueth et al., 2022; Wang, 2023) and three for LBW (Wallace et al., 2013b; Barrett et al., 2018; Wang, 2023). Key findings are summarized in Table 2.

#### AL and PTB

**Figure 2:**
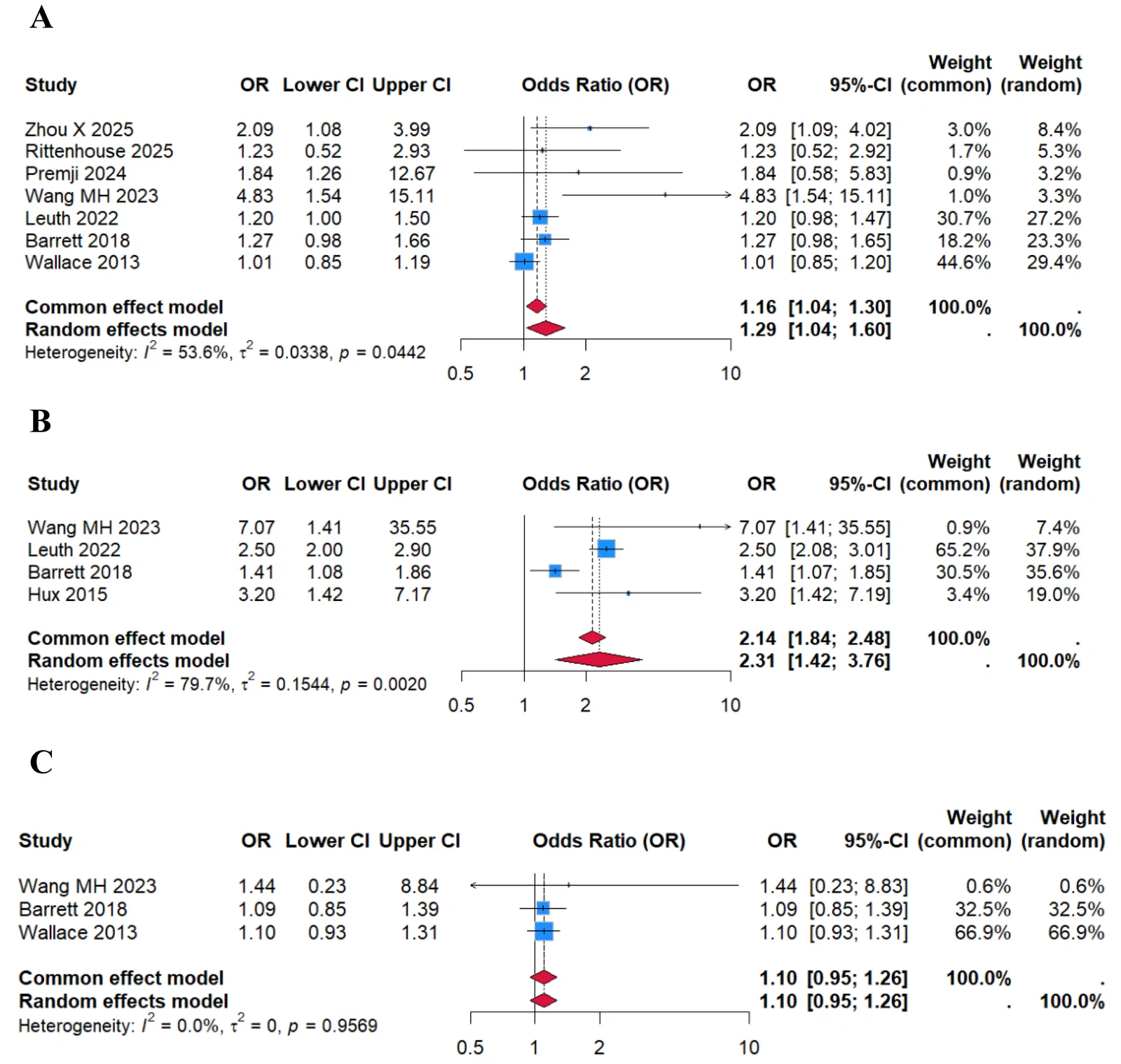
Forest plot of the association between prenatal AL and APOs. (A) PTB, (B) PE, (C) LBW.

Seven prospective studies examined the association between AL and PTB, involving a total of 8,678 pregnant women. The heterogeneity test indicated substantial variance among the included studies for PTB (*I* ^2^ = 53.6%, *P* = 0.044). The random-effects model was primarily utilized, which demonstrated a statistically significant association between high AL levels and the risk of PTB (random-effects model: OR = 1.29, 95% CI [1.04–1.60]) (Fig. 2A).

**Table 1:** Basic characteristics of included studies and results of bias risk assessment.

**Study**	**Country**	**Design**	**Sample** **size**	**Gestational period** (week)	**Method of AL score construction**	**Biomarker of AL score construction**	**Outcomes**	**Adjusted covariates**	**NOS a** **ss** **essment**
Wallace et al. (2013b)	United States	Cohort	866	Preconception	Dichotomization of biomarkers, distribution-based, then summed across markers, then summed.	SBP, DBP, total cholesterol, HDL, LDL, triglycerides, glucose, insulin and waist circumference	LBW/PTB	Age, smoking during pregnancy, year of BHS examination and years between examination and conception	7
Hux & Roberts (2015)	United States	Case-control	117	First trimester (9.1 ± 2.5)	Within-system dichotomization of biomarkers, based on distributions of a nationally representative sample. Proportion of high-risk biomarkers within each system then summed	SBP, DBP, pulse pressure, pre-pregnancy BMI, total cholesterol, HDL, non-fasting triglycerides, CRP and interleukin-6	PE	Gestational age at delivery	9
Barrett et al. (2018)	United States	Cohort	836	Preconception	Dichotomization of biomarkers, distribution-based, then summed across markers, then summed.	BMI, waist-to-hip ratio, SBP, DBP, dehydroepiandrosterone sulfate, HDL, LDL, triglycerides, CRP and HOMA score^a^	LBW/PTB/PE	Age, race, drinking habits, smoking habits and income	7
Lueth et al. (2022)	United States	Cohort	4,266	First trimester (6^+0^-13^+0^)	Dichotomization of biomarkers, distribution-based, then summed across markers. Then summed. Converted into a dichotomous variable (high AL≥4)	SBP, DBP, cholesterol, LDL, HDL, hsCRP, BMI, urine creatinine, urine albumin, triglycerides, insulin and glucose	SGA/PTB/PE/ Stillbirth	Age, number of pregnancies, smoking habits, bleeding during early pregnancy and type of health insurance	8
Wang et al. (2023)	China	Cohort	354	Third trimester (30^+0^-34^+0^)	Dichotomization of biomarkers, distribution-based, then summed across markers, then summed.	BMI, SBP, DBP, waist circumference, total cholesterol, HDL, glucose and CRP	LBW/PTB/PE/ Macrosomia	Age, occupation, educational level, income, history of miscarriage, anemia during pregnancy and GDM	9
Premji et al. (2025)	Pakistan	Cohort	1,567	Second trimester (14)	Conversion of biomarkers to z-scores, then summed.	cortisol, glycosylated hemoglobin, CRP, total cholesterol, SBP and DBP	PTB	Multiple pregnancies	8
Rittenhouse et al. (2025)	Zambia	Cohort	152	Second trimester (24)	Conversion of biomarkers to z-scores, then summed.	SBP, DBP, pulse, BMI, upper arm circumference, total cholesterol, HDL, triglycerides, hemoglobin, albumin, CRP, cortisol, progesterone, estradiol and 25-hydroxyvitamin D	PTB	Age, reproductive history and gestational weeks at enrollment	7
Zhou et al. (2025)	China	Cohort	637	Second trimester (20^+0^-24^+0^)	Dichotomization of biomarkers, distribution-based, then summed across markers, then summed.	SBP, DBP, total cholesterol, triglycerides, BMI, fasting blood glucose, hsCRP, albumin, creatinine and cortisol	PTB	Age, occupation, educational level, weight, fetal rate, abnormal reproductive history, family history of hypertension and family history of diabetes	8

**Notes.**

Abbreviations CRP C-reactive protein DBP diastolic blood pressure HDL high-density lipoprotein hsCRP high sensitivity C-reactive protein HOMA homeostasis model assessment LBW low birth weight LDL low-density lipoprotein PE preeclampsia PTB preterm birth SBP systolic blood pressure SGA small for gestational age

a HOMA=insulin*glucose/40.

**Table 2:** Association between AL and APOs.

Adverse pregnancy outcomes	Heterogeneity							Effect size		
	K^a^	N^b^	Q	*P*	*I* ^2^	*T* ^2^		*OR*	95% *CI*	*P* ^c^
PTB	7	8,678	12.93	0.044	53.6	0.034		1.29	1.04–1.60	**0.022**
PE	4	5,573	14.80	0.002	79.7	0.154		2.31	1.42–3.76	**<0.001**
LBW	3	2,056	0.09	0.960	0.0	0.0		1.10	0.95–1.26	0.189
SGA	1	4,266	Not applicable (single study)^d^					0.80	0.62–1.03	–^e^
Macrosomia	1	354	Not applicable (single study)^d^					2.87	1.39–5.91	**0.004**
Stillbirth	1	4,266	Not applicable (single study)^d^					2.60	0.80–8.30	–^e^

**Notes.**

a K = number of studies.

b N = total number of participants.

c *P*-values(two-tailed):Statistically significant(*P* <0.05) in **bold**.

d SGA, macrosomia and stillbirth are descriptive results from single studies rather than meta-analyses.

e The original study did not provide extractable *P*-values.

To further explore sources of high heterogeneity, we conducted subgroup analyses (Table 3). Stratification by gestational period revealed that high AL levels in the second trimester (OR = 1.74, 95% CI [1.08–2.80]) and the third trimester (OR = 4.83, 95% CI [1.54–15.11]) were positively associated with PTB (*P* = 0.036). Additionally, analyses based on AL biomarker composition indicated that high AL levels incorporating immune system indicators were associated with PTB (OR = 1.30, 95% CI [1.12–1.51], *P* = 0.028) (Table S1 and Fig. S1). However, it must be emphasized that these subgroup analysis estimates are purely exploratory and some findings were based on only a single study with a notably wide confidence interval, necessitating cautious interpretation.

#### AL and PE

The results of a meta-analysis of four studies demonstrated that high AL was significantly associated with a higher likelihood of PE (random-effects model: OR = 2.31, 95% CI [1.42–3.76]), albeit with substantial heterogeneity (*I* ^2^ = 79.7%, *P* = 0.002) (Fig. 2B).

Exploratory subgroup analyses were also conducted based on country, gestational period, and the inclusion of neuroendocrine system biomarkers (Table 4). Regarding the gestational period, the pooled OR for pre-pregnancy AL levels and PE risk was 1.41 (95% CI [1.07–1.85]), whereas studies assessing AL in the first trimester reported an OR of 2.53 (95% CI [2.11–3.03]), and the single study assessing AL in the third trimester reported an OR of 7.07 (95% CI [1.41–35.55]) (*P* < 0.001), this subgroup difference should be treated strictly as an exploratory observation, given that it relies on a single study with a very wide confidence interval. Furthermore, in studies where the AL index did not include neuroendocrine biomarkers, high AL was strongly associated with an increased risk of PE (OR = 2.56, 95% CI [2.14–3.07]). Conversely, the single study that included neuroendocrine biomarkers demonstrated a comparatively attenuated, yet still significant, positive association with PE (OR = 1.41, 95% CI [1.07–1.85]) (*P* < 0.001), which similarly requires cautious interpretation (Table S2 and Fig. S2).

**Table 3:** Subgroup analyses of the association between prenatal AL and PTB.

**Variable**	**Subgroup**	**Studies**	***OR*** ** (95%** ***CI*** **)**	***I*** ^**2**^ **(%)**	***P*** ^a^ ** for subgroup differences**
Country	United States	3	1.13 (0.98–1.30)	27.7	0.134
	China	2	2.77 (1.28–6.04)	36.0	
	Pakistan	1	1.84 (0.58–5.83)	–	
	Zambia	1	1.23 (0.52–2.92)	–	
Gestational age	Preconception	2	1.11 (0.89–1.38)	51.5	**0.036**
	First trimester	1	1.20 (0.98–1.47)	–	
	Second trimester	3	1.74 (1.08–2.80)	0.0	
	Third trimester	1	4.83 (1.54–15.11)	–	
AL scoring methods	Dichotomization	5	1.30 (1.01–1.66)	67.5	0.805
	Z-scores	2	1.42 (0.71–2.84)	0.0	
Immune system	Included	6	1.30 (1.12–1.51)	38.4	**0.028**
	Excluded	1	1.01 (0.85–1.20)	–	
Neuroendocrine system	Included	4	1.39 (1.07–1.81)	0.0	0.862
	Excluded	3	1.49 (0.72–3.10)	75.5	

**Notes.**

a *P*-values (two-tailed): Statistically significant (*P* <0.05) in bold.

**Table 4:** Subgroup analyses of the association between prenatal AL and PE.

**Variable**	**Subgroup**	**Studies**	**OR (95% CI)**	***I*** ^**2**^ **(%)**	***P*** ^a^ ** for subgroup differences**
Country	United States	3	2.10 (1.32–3.34)	84.2	0.156
	China	1	7.07 (1.41–35.55)^b^	–	
Gestational age	Preconception	1	1.41 (1.07–1.85)^b^	–	**<0.001**
	First trimester	2	2.53 (2.11–3.03)	0.00	
	Third trimester	1	7.07 (1.41–35.55)^b^	–	
Neuroendocrine system	Included	1	1.41 (1.07–1.85)^b^	–	**<0.001**
	Excluded	3	2.56 (2.14–3.07)	0.00	

**Notes.**

a *P*-values (two-tailed): Statistically significant (*P* <0.05) in bold.

b Derives from a single study, advising cautious interpretation.

#### AL and LBW

Three studies investigated the association between AL levels and LBW. The random-effects model results indicated that current evidence does not support a significant association between AL and LBW (random-effects model: OR = 1.10, 95% CI [0.95–1.26]) with minimal heterogeneity (*I*^2^ = 0%, *P* = 0.957).

#### AL and other APOs

Evidence for SGA, stillbirth and macrosomia was limited to single studies. Lueth et al. (2022) analyzed the nuMoM2b cohort and found no statistically significant association between high AL levels in early pregnancy and the likelihood of stillbirth or SGA (OR = 2.60, 95% CI [0.80–8.30]; OR = 0.80, 95% CI [0.62–1.03]). Furthermore, Wang investigated the impact of high AL levels in late pregnancy on maternal and fetal outcomes in a study conducted in Anhui, China, reporting that high AL levels were linked to higher odds of macrosomia in late pregnancy (aOR = 2.87, 95% CI [1.39–5.91]).

### Publication bias and sensitivity analysis

**Figure 3:**
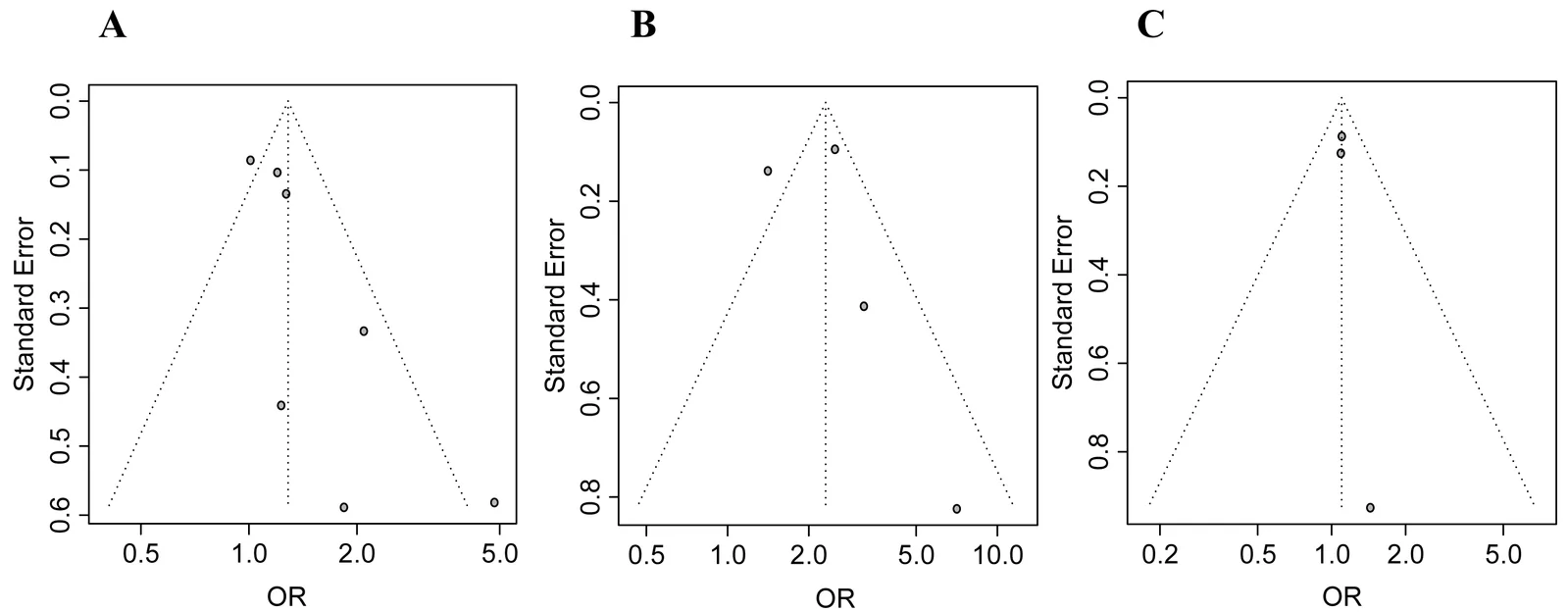
The funnel plot and trim-and fill of the association between prenatal AL and APOs. (A) Funnel plot of PTB, (B) funnel plot of PE, (C) funnel plot of LBW.

Given the small number of included studies (<10) for each outcome, we conducted an exploratory analysis of potential publication bias using funnel plots. Visual inspection of the funnel plots suggested potential asymmetry for PTB, PE, and LBW (Fig. 3). Due to the limited number of studies, these publication bias assessments should be interpreted with caution.

As a sensitivity analysis to further explore our primary findings, we also applied a fixed-effects model. The results from this approach were generally consistent with the random-effects estimates. Specifically, the fixed-effects models continued to suggest a potential association between high AL levels and increased risks of PTB (OR = 1.16, 95% CI [1.04–1.30]) and PE (OR = 2.14, 95% CI [1.84–2.48]). Conversely, the estimate for LBW remained statistically non-significant (OR = 1.10, 95% CI [0.95–1.26]).

**Figure 4:**
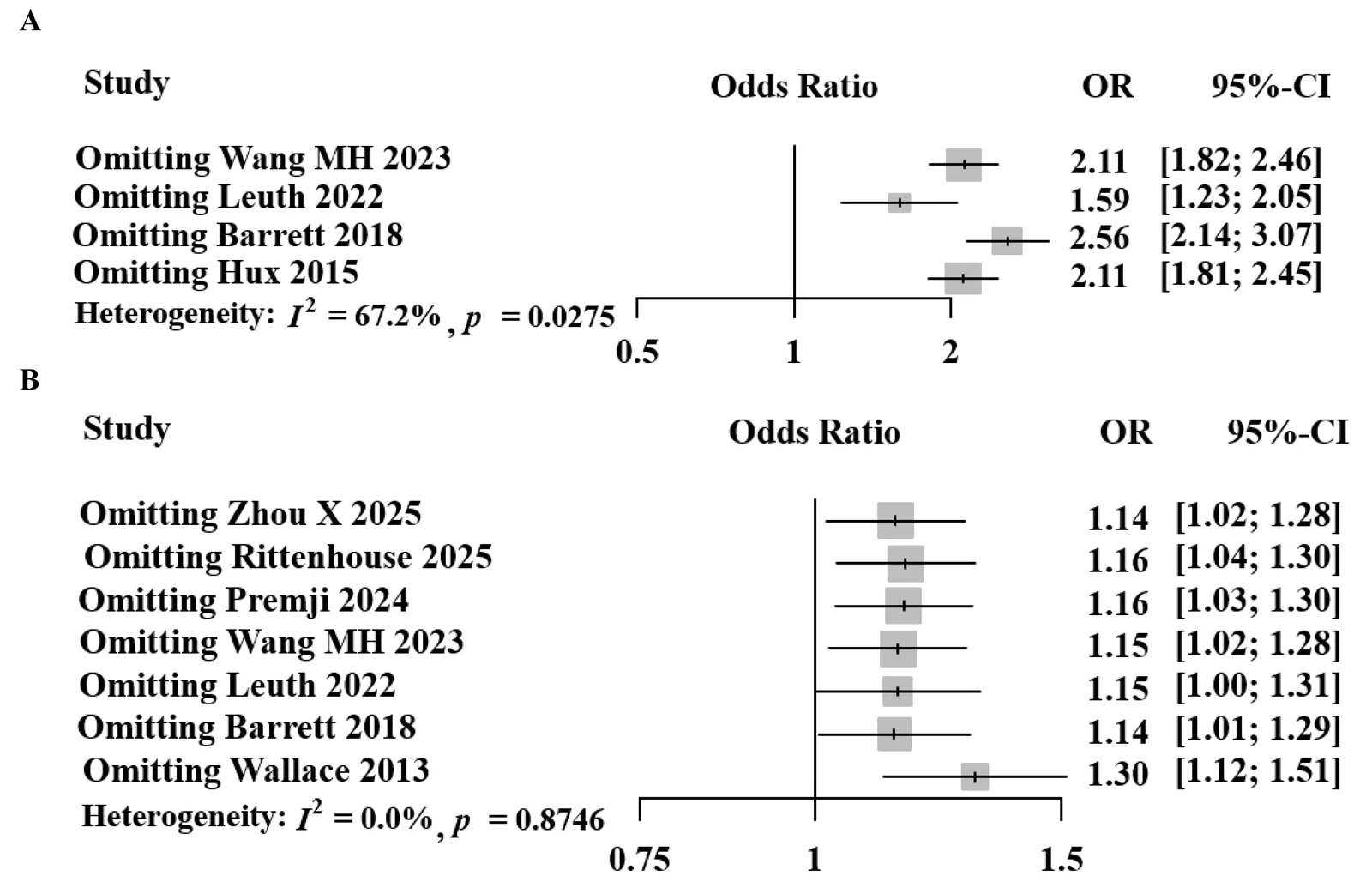
Leave one out of the association between prenatal AL and APOs. (A) Leave one out of PTB, (B) leave one out of PE.

To evaluate the influence of individual studies on the overall pooled estimates, a leave-one-out sensitivity analysis was conducted for the PTB and PE outcomes. The results demonstrated that sequentially omitting any single study did not materially alter the pooled effect sizes or their statistical significance. The recalculated pooled ORs ranged from 1.14 (95% CI [1.01–1.29]) to 1.30 (95% CI [1.12–1.51]) for PTB, and from 1.59 (95% CI [1.23–2.05]) to 2.56 (95% CI [2.14–3.07]) for PE. These results suggest that the primary meta-analysis estimates are generally stable within the observed ranges (Figs. 4A, 4B).

## Discussion

This systematic review and meta-analysis represent the first comprehensive evaluation of the association between prenatal AL and multiple APOs. Our findings demonstrate that elevated prenatal AL is significantly associated with a higher likelihood of PTB and PE, with exploratory subgroup analyses suggesting that the gestational period and biomarker composition may influence these associations.

Previous studies have found associations between prenatal stress biomarkers and APOs. García-Blanco et al. (2017) discovered that elevated salivary cortisol was a predictor of PTB, while meta-analytic evidence has revealed associations between inflammatory markers (interleukin-6, CRP) and heightened risks of PTB and PE (Admas, Teoh & Chonu, 2025). However, reliance on isolated biomarkers presents certain limitations: cortisol is sensitive to environmental changes and fluctuates rapidly, rendering single measurements inadequate proxies for chronic stress (Hoffman et al., 2016). Concurrently, pregnancy-induced physiological elevations in inflammatory markers obscure the differentiation between pathological and adaptive inflammation (Mor & Cardenas, 2010). Consequently, mono-dimensional biomarkers provide limited information about stress pathways, restricting both predictive utility and mechanistic interpretation. In contrast, the AL index synthesizes multidimensional biomarkers across neuroendocrine, immune, metabolic, and cardiovascular systems, providing a holistic quantification of chronic stress burden, offering a theoretically broader biological framework for understanding APOs risk. Notably, while our subgroup analyses suggested potential variations in the AL-PTB association when immune or neuroendocrine markers were included in the AL index, these findings remain strictly exploratory. Given that certain reference categories contained only a single study, there is currently insufficient evidence to draw definitive conclusions from these observed differences.

In the analysis of PTB, our findings indicated that elevated prenatal AL levels were associated with the occurrence of PTB. Specifically, subgroup analyses indicated that higher AL measured during the second and third trimester correlated with an elevated risk of PTB. However, these trimester-specific estimates must be interpreted with extreme caution, as they are strictly exploratory and, in several instances, are based on only one study with wide confidence intervals. One plausible biological hypothesis is that elevated AL may be associated with dysregulation of both the HPA axis and sympatho-adrenomedullary (SAM) axis. It is postulated that dysregulation of the HPA axis may increase cortisol secretion, which is hypothesized to potentiate placental corticotropin-releasing hormone (CRH) release and inflammatory pathways that have been hypothesized to promote uterine contractility; simultaneously, dysregulation of the SAM axis may increase catecholamine release, potentially enhancing myometrial sensitivity to oxytocin, which could contribute to vasoconstriction (compromising placental perfusion), and possibly accelerating cervical remodeling (Hoffman et al., 2016; García-Blanco et al., 2017; Mayne et al., 2025; Admas, Teoh & Chonu, 2025). Collectively, these findings are consistent with the hypothesis that dysregulation of the HPA and SAM axes may represent one of several biological pathways linking elevated AL with PTB, although these pathways remain purely theoretical and cannot be established as causal mechanisms from the present observational evidence. The high heterogeneity observed in this study may be partially attributable to differences in country demographics and AL assessment methods among various studies. Subgroup analyses suggested that the timing of AL assessment during pregnancy and the composition of biomarkers may partially explain the variability in the observed associations with PTB. However, these subgroup findings should be interpreted with caution because several subgroups included only a limited number of studies, resulting in imprecise effect estimates with wide confidence intervals. Therefore, the observed sources of heterogeneity should be considered exploratory.

Our results integrate existing evidence, indicating an association between prenatal AL and PE (Hux & Roberts, 2015; Barrett et al., 2018; Lueth et al., 2022; Wang et al., 2023). In exploratory subgroup analyses, we observed that after incorporating neuroendocrine indicators into the AL index, the effect size of the association between AL and PE may have attenuated. Due to the small number of studies contributing to these subgroups, it remains highly speculative to link broader multisystem AL indices to distinct biological mechanisms. If a true association exists, one potential explanation is that HPA/SAM dysregulation might be a contributing factor, which has been theoretically linked to endothelial dysfunction, pro-inflammatory states, and hypertension potentially through alterations in catecholamine signaling (Ahmadian et al., 2020; Ali et al., 2024). The temporal specificity of this association may reflect dynamic physiological adaptations: neuroendocrine markers exhibit gestational-period-dependent fluctuations in healthy pregnancies, and pre-pregnancy AL assessments might only capture baseline states (Orta et al., 2017). Furthermore, neuroendocrine compensatory mechanisms could partially mitigate AL impacts in earlier gestation (Glynn, Davis & Sandman, 2013). However, it is important to acknowledge that the operationalization of AL varies substantially across the included studies. Discrepancies in the types and absolute numbers of biomarkers included in the AL algorithms introduce significant heterogeneity, which may confound the pooled effect sizes and limit direct cross-study comparisons. Meanwhile, these subgroup analyses were based on relatively few studies, resulting in wide confidence intervals and limiting the precision of the estimated effects.

Intriguingly, while AL showed no significant association with LBW, SGA, or stillbirth, it was positively correlated with macrosomia risk. This counter prevailing hypotheses suggesting AL restricts fetal growth (Wallace et al., 2013a; Admas, Teoh & Chonu, 2025). One possible explanation is that pregnant women with high AL levels may exhibit changes in behavioral patterns, such as increased intake of high-sugar, high-fat foods and reduced physical activity, which indirectly affect fetal growth (Matvienko-Sikar et al., 2025). However, there is currently only one study on AL and macrosomia. Thus, these results require cautious interpretation, and more cohort studies are needed for validation.

This study synthesized existing evidence through systematic reviews and meta-analyses, suggested that the gestational period may be a source of heterogeneity in prenatal AL, and explored the association between AL and various pregnancy outcomes, providing a basis for future research directions. However, the current literature presents several limitations. Firstly, the concept of AL in the included studies was constrained to a perspective that solely focused on biomarkers. This approach relied solely on downstream cumulative physiological scores and failed to account for critical upstream psychosocial stressors, such as transgenerational trauma, childhood adversity, and chronic socioeconomic deprivation. Therefore, it could only provide partial information about maternal stress exposure. Meanwhile, some biomarkers such as blood pressure, BMI, glucose, and lipids are themselves established predictors of adverse pregnancy outcomes. Consequently, the observed associations may partly reflect aggregation of conventional obstetric risk factors rather than independent effects of chronic stress biology. Secondly, the lack of consensus on AL assessment introduces significant heterogeneity in current research. This is manifested in varying indicator quantities, different threshold standards, incomplete coverage of biological systems, and diverse construction methods of statistical indices, all of which reduce the comparability of the results from different studies. Thirdly, the confounding factors included in each study are different, which may be the cause of the heterogeneity among the results. Additionally, some studies also have unmeasured confounding factors that have not been adjusted for. Finally, the evidence for certain clinical endpoints is still extremely limited. Outcomes such as SGA, stillbirth, and macrosomia are only reported by a single study, so these findings should be interpreted with caution. The majority of the included studies come from the United States, which further limits the generalizability and applicability of these findings in other geographical regions and among different populations. Future research should focus on conducting more high-quality prospective maternal-fetal cohort studies to further elucidate the associations between AL levels at different stages of pregnancy and maternal-fetal outcomes.

## Conclusion

Prenatal AL is associated with APOs, particularly PE and PTB, and the timing of the gestational period for AL assessment and the biomarker composition may affect the strength of the above association to a certain extent, although these variations remain entirely exploratory. Future studies should develop prospective longitudinal cohort designs to conduct repeated dynamic measurements of AL throughout pregnancy, in order to establish a scientific and standardized AL evaluation framework. Given the current absence of standardized AL indices, validated clinical thresholds, and specific intervention trials, prenatal AL assessment is not yet applicable in routine clinical practice. Ultimately, rigorously designed studies are required to determine whether AL can be validated as an independent and clinically feasible risk predictor for adverse pregnancy outcomes as a future research direction.

##  Supplemental Information

10.7717/peerj.21756/supp-1 Supplemental Information 1 PRISMA checklist

10.7717/peerj.21756/supp-2 Supplemental Information 2 MOOSE Checklist

10.7717/peerj.21756/supp-3 Supplemental Information 3 Search strategy and supplemental tables and figures = Search Strategy

10.7717/peerj.21756/supp-4 Supplemental Information 4 Raw data

## Abbreviations

AL: Allostatic Load
APOs: Adverse Pregnancy Outcomes
BMI: Body Mass Index
CI: Confidence Interval
CNKI: China National Knowledge Infrastructure
CRH: Corticotropin-Releasing Hormone
CRP: C-reactive Protein
DBP: Diastolic Blood Pressure
HDL: High-Density Lipoprotein
HPA: Hypothalamic-Pituitary-Adrenal
hsCRP: high sensitivity C-Reactive Protein
LBW: Low Birth Weight
LDL: Low-Density Lipoprotein
NOS: Newcastle-Ottawa Scale
OR: Odds Ratio
PE: Preeclampsia
PRISMA: Preferred Reporting Items for Systematic Reviews And Meta-Analyses
PROSPERO: International Prospective Register of Systematic Reviews
PTB: Preterm Birth
SAM: Sympatho-Adrenomedullary
SBP: Systolic Blood Pressure
SGA: Small for Gestational Age
VIP: China Science and Technology Journal Database (VIP)

## Additional Information and Declarations

### Data Availability

The following information was supplied regarding data availability: This is a Systematic review/Meta-analysis.
