# Supplementary material for: Prenatal allostatic load and adverse pregnancy outcomes: a systematic review and meta-analysis
Source: PeerJ. 2026 Sep 24;14:e21756. doi: 10.7717/peerj.21756 (PMC13616195; doi:10.7717/peerj.21756)
Supplement: Supplementary file 2 [file peerj-14-21756-s002.pdf]

MOOSE (Meta-analyses Of Observational Studies in Epidemiology) Checklist

A reporting checklist for Authors, Editors, and Reviewers of Meta-analyses of Observational Studies. You must report the page number in your manuscript where you consider each of the items listed in this checklist. If you have not included this information, either revise your manuscript accordingly before submitting or note N/A.

Reporting Criteria	Reported (Yes/No)	Reported on Page No.
**Reporting of Background**		
Problem definition	Yes	2
Hypothesis statement	Yes	2
Description of Study Outcome(s)	Yes	2
Type of exposure or intervention used	Yes	2
Type of study design used	Yes	2
Study population	Yes	2
**Reporting of Search Strategy**		
Qualifications of searchers (eg, librarians and investigators)	Yes	3
Search strategy, including time period included in the synthesis and keywords	Yes	2-3
Effort to include all available studies, including contact with authors	Yes	3
Databases and registries searched	Yes	2
Search software used, name and version, including special features used (eg, explosion)	Yes	3
Use of hand searching (eg, reference lists of obtained articles)	Yes	3
List of citations located and those excluded, including justification	Yes	2
Method for addressing articles published in languages other than English	Yes	2
Method of handling abstracts and unpublished studies	Yes	2
Description of any contact with authors	Yes	3
**Reporting of Methods**		
Description of relevance or appropriateness of studies assembled for assessing the hypothesis to be tested	Yes	3-5
Rationale for the selection and coding of data (eg, sound clinical principles or convenience)	Yes	3
Documentation of how data were classified and coded (eg, multiple raters, blinding, and interrater reliability)	Yes	3
Assessment of confounding (eg, comparability of cases and controls in studies where appropriate	Yes	3

Reporting Criteria	Reported (Yes/No)	Reported on Page No.
Assessment of study quality, including blinding of quality assessors; stratification or regression on possible predictors of study results	Yes	3
Assessment of heterogeneity	Yes	3
Description of statistical methods (eg, complete description of fixed or random effects models, justification of whether the chosen models account for predictors of study results, dose-response models, or cumulative meta-analysis) in sufficient detail to be replicated	Yes	3
Provision of appropriate tables and graphics	Yes	4-13
**Reporting of Results**		
Table giving descriptive information for each study included	Yes	6-8
Results of sensitivity testing (eg, subgroup analysis)	Yes	9-13
Indication of statistical uncertainty of findings	Yes	9
**Reporting of Discussion**		
Quantitative assessment of bias (eg, publication bias)	Yes	10-12
Justification for exclusion (eg, exclusion of non–English-language citations)	No	
Assessment of quality of included studies	Yes	6-7
**Reporting of Conclusions**		
Consideration of alternative explanations for observed results	Yes	14
Generalization of the conclusions (ie, appropriate for the data presented and within the domain of the literature review)	Yes	14
Guidelines for future research	Yes	15
Disclosure of funding source	Yes	16

Once you have completed this checklist, please save a copy and upload it as part of your submission. DO NOT include this checklist as part of the main manuscript document. It must be uploaded as a separate file.
