# Supplementary material for: Prenatal allostatic load and adverse pregnancy outcomes: a systematic review and meta-analysis
Source: PeerJ. 2026 Sep 24;14:e21756. doi: 10.7717/peerj.21756 (PMC13616195; doi:10.7717/peerj.21756)
Supplement: Supplementary file 3 [file peerj-14-21756-s003.docx]

**Supplementary 3：**

**框1　PubMed检索策略**

#1 Allostasis [Mesh Terms]

#2 Allosatatic load[Title/Abstract]

#3 Allostasesl[Title/Abstract]

#4 Allostatic Loads[Title/Abstract]

#5 Load, Allostatic[Title/Abstract])

#6 Loads, Allostatic[Title/Abstract]

#7 #1 OR #2 OR #3 OR #4 OR #5 OR #6

#8 Pregnancy Complications [Mesh Terms]

#9 Complication, Pregnancy[Title/Abstract]

#10 Pregnancy Complication[Title/Abstract]

#11 Complications, Pregnancy[Title/Abstract]

#12 Outcome, Adverse Birth[Title/Abstract]

#13 Adverse Birth Outcomes[Title/Abstract]

#14 Adverse Birth Outcome[Title/Abstract]

#15 Birth Outcome, Adverse[Title/Abstract]

#16 preterm birth[Title/Abstract]

#17 low birth weight[Title/Abstract]

#18 preeclampsia[Title/Abstract]

#19 pregnancy[Title/Abstract]

#20 #8 OR #9 OR ##10 OR #11 OR #12 OR #13 OR #14 OR #15 OR #16 OR #17 OR #18 OR #19

**Supplementary Table 1. Studies contributing to each subgroup pooled estimate of PTB**

| **Variable** | **Subgroup level** | **No. of studies** | **Study ID (Author,Year)** | **Estimate type** |
| --- | --- | --- | --- | --- |
| Country | United States | 3 | Wallace et al., 2013b; Barrett et al., 2018; Leuth et al., 2022. | Pooled effect size |
|  | China | 2 | Wang MH et al., 2023; Zhou X et al., 2025. | Pooled effect size |
|  | Pakistan | 1 | Premji et al., 2024. | Single-study descriptive estimate |
|  | Zambia | 1 | Rittenhouse et al., 2025. | Single-study descriptive estimate |
| Gestational age | Preconception | 2 | Wallace et al., 2013b; Barrett et al., 2018. | Pooled effect size |
|  | First trimester | 1 | Leuth et al., 2022. | Single-study descriptive estimate |
|  | Second trimester | 3 | Premji et al., 2024; Rittenhouse et al., 2025; Zhou X et al., 2025. | Pooled effect size |
|  | Third trimester | 1 | Wang MH et al., 2023 | Single-study descriptive estimate |
| AL scoring methods | Dichotomization | 5 | Wallace et al., 2013b; Barrett et al., 2018; Leuth et al., 2022; Wang MH et al., 2023; Zhou X et al., 2025. | Pooled effect size |
|  | Z-scores | 2 | Premji et al., 2024; Rittenhouse et al., 2025; | Pooled effect size |
| Immune system | Included | 6 | Barrett et al., 2018; Leuth et al., 2022; Wang MH et al., 2023; Premji et al., 2024; Rittenhouse et al., 2025; Zhou X et al., 2025. | Pooled effect size |
|  | Excluded | 1 | Wallace et al., 2013b. | Single-study descriptive estimate |
| Neuroendocrine system | Included | 4 | Barrett et al., 2018; Premji et al., 2024; Rittenhouse et al., 2025; Zhou X et al., 2025. | Pooled effect size |
|  | Excluded | 3 | Wallace et al., 2013; Leuth et al., 2022; Wang MH et al., 2023; | Pooled effect size |

**Supplementary Table 2. Studies contributing to each subgroup pooled estimate of PE**

| **Variable** | **Subgroup level** | **No. of studies** | **Study ID (Author,Year)** | **Estimate type** |
| --- | --- | --- | --- | --- |
| Country | United States | 3 | Hux et al., 2015; Barrett et al., 2018; Leuth et al., 2022. | Pooled effect size |
|  | China | 1 | Wang MH et al., 2023. | Single-study descriptive estimate |
| Gestational age | Preconception | 1 | Barrett et al., 2018. | Single-study descriptive estimate |
|  | First trimester | 2 | Hux et al., 2015; Leuth et al., 2022. | Pooled effect size |
|  | Third trimester | 1 | Wang MH et al., 2023. | Single-study descriptive estimate |
| Neuroendocrine system | Included | 1 | Barrett et al., 2018. | Single-study descriptive estimate |
|  | Excluded | 3 | Hux et al., 2015; Leuth et al., 2022; Wang MH et al., 2023. | Pooled effect size |

**Supplementary Figure 1. Subgroup analysis of AL and PTB**

Supplementary Figure 1-A. Subgroup analysis of AL and PTB based on the country

Supplementary Figure 1-B. Subgroup analysis of AL and PTB based on the gestional age

Supplementary Figure 1-C. Subgroup analysis of AL and PTB based on the AL methods scoring

Supplementary Figure 1-D. Subgroup analysis of AL and PTB based on the immune system

Supplementary Figure 1-E. Subgroup analysis of AL and PTB based on the neuroendocrine system

**Supplementary Figure 2. Subgroup analysis of AL and PE**

Supplementary Figure 2-A. Subgroup analysis of AL and PE based on the country

Supplementary Figure 2-B. Subgroup analysis of AL and PE based on the gestational age

Supplementary Figure 2-C. Subgroup analysis of AL and PE based on the neuroendocrine system
